# Comparison of Postoperative Outcome in Children Undergoing Inguinal Hernia Repair Using Regional With General Versus General Anesthesia Alone: A Single Center Study

**DOI:** 10.7759/cureus.37382

**Published:** 2023-04-10

**Authors:** Omar M Kabbani, Khaled A Alhabdan, Abdulaziz Y Almahbub, Nasib M Kabbani, Husam I Ardah, Ahmed Haroun M Mahmoud

**Affiliations:** 1 College of Medicine, King Saud Bin Abdulaziz University for Health Sciences, Riyadh, SAU; 2 Department of Anesthesia, King Abdulaziz Medical City, Ministry of the National Guard Health Affairs, Riyadh, SAU; 3 Biostatistics and Epidemiology, King Abdullah International Medical Research Center, Riyadh, SAU; 4 Medical Education, College of Medicine, King Saud Bin Abdulaziz University for Health Sciences, Riyadh, SAU; 5 Research and Development, King Abdullah International Medical Research Center, Riyadh, SAU; 6 Pediatric Anesthesiology, King Abdullah Specialised Children Hospital, Riyadh, SAU

**Keywords:** anesthesia, general anesthesia, regional, pediatrics, inguinal hernia

## Abstract

Background: Inguinal hernia repair is one of the most common general surgical procedures. It has been performed under local, regional, or general anesthesia. We hypothesized that using regional plus general anesthesia rather than general anesthesia alone would improve outcomes in neonates and pediatric patients undergoing hernia repair.

Methods: This is a retrospective cohort study, including all pediatric patients who underwent inguinal hernia repair from 2015-2021. We divided patients into two groups. The first group was labeled "general anesthesia" (GA), while the second group was labeled "combined general and regional anesthesia" (GA+RA). We compared both groups in terms of demographic data, intraoperative outcome variables, and postoperative outcome variables.

Results: 212 children fulfilled the study criteria, with 57 in the GA group and 155 in the GA+RA group. Demographic and preoperative data were comparable between both groups except for age, which was 60.3±49.4 months in the GA group versus 26.7±33.13 months in the GA+RA group (p<.0001). Outcome variables demonstrated statistically significant improvement in postoperative pain occurrence, length of hospital stay, incidence of bradycardia, and need for mechanical ventilation in the GA+RA group in comparison to the GA group with P values of 0.031, 0.02, 0.005, and 0.02, respectively.

Conclusion: Using regional and general anesthesia techniques rather than general anesthesia alone is associated with a decrease in postoperative pain, length of hospital stay, incidence of bradycardia, and need for mechanical ventilation. Further studies are still warranted to validate our conclusions.

## Introduction

Hernias occur when part of the bowel or peritoneum protrudes through a gap in the abdominal wall that has a weak musculoaponeurotic barrier [1]. Types of hernias include inguinal, femoral, and umbilical hernias. Pediatric hernia is a common condition with an estimated prevalence of 5% in the general population, with inguinal hernias being the most common type of hernias [2]. Factors that may contribute to inguinal hernias are prematurity, undescended testicles, a family history of hernias, and cystic fibrosis [3].

The majority of pediatric inguinal hernias require surgical repair to prevent the development of complications, such as incarceration or strangulation. Therefore, it is considered one of the most common pediatric surgical operations [4]. Many of the postoperative complications encountered after hernia repair are linked to the anesthesia rather than the herniorrhaphy itself, especially when using general anesthesia. Examples of these complications include postoperative ileus, urinary retention, postoperative hypotension, nausea, and vomiting [5].

Hernia surgeries in the past have primarily been done under general anesthesia [6]. The use of a combination of regional and general anesthesia, however, may result in improved postoperative care and fewer adverse effects from general anesthesia, according to recent studies [7,8]. In order to prevent or reduce pain, regional anesthesia involves injecting a local anesthetic into a peripheral nerve [9]. This prevents the transmission of pain signals to the brain. This technique may have the benefit of requiring less intraoperative anesthetic medications with fewer postoperative side effects. The choice of anesthetic technique is influenced by a number of variables, such as the anesthesiologist's skills and desire for doing a regional block, institutional practice, the acceptance of the patient's legal guardian, and the existence of regional anesthesia contraindications [10]. The existence of spinal abnormalities or coagulopathy is an example that limits the use of regional anesthesia and promotes the only use of general anesthesia in these situations [11].

The benefits of combining general and regional anesthesia have not been adequately studied in the pediatric clinical setting. In children, there is a knowledge gap and a paucity of information regarding the relevance and benefits of combining regional and general anesthesia for pediatric hernia repair over general anesthesia alone [12]. Systemic anesthetics and opioids have been linked to neurological and cardiorespiratory problems in preterm babies and young infants. Utilizing fewer general anesthetics and opioids may minimize morbidity and improve postoperative outcomes.

To explore the potential benefits of combining GA and RA in comparison to GA alone for pediatric hernia repair, we conducted this retrospective study to answer the question of whether combining regional and general anesthesia is better than general anesthesia alone for postoperative care and co-morbidities following inguinal hernia repair in children.

## Materials and methods

This study is a retrospective cohort study conducted between 2015 and 2021 in a tertiary specialized children's hospital. The institutional review board of King Abdullah International Medical Research Center (KAIMRC) approved the study (RYD-22-419812-143473). The study included all pediatric patients from birth to 14 years of age who had open or laparoscopic inguinal hernia repair under regional and general or general anesthesia alone. Both inpatients and ambulatory care patients were included in our study. We excluded patients who had other procedures as a primary operation not consistent with inguinal hernia repair except for minimal surgery done in conjunction with hernia repair, such as circumcision. Furthermore, patients who were unstable hemodynamically prior to surgery, patients with chronic renal failure receiving renal replacement therapy, and those who had life-threatening conditions were also excluded from the study.

We reviewed the data in the computerized digital medical records system (Best Care System). We extracted all data, which included demographic data, perioperative data, and postoperative outcome variables including postoperative pain and analgesic requirement, time spent in the post-anesthesia care unit (PACU), scrotal edema, behavioral changes postoperatively, bradycardia or tachycardia, hypotension, mechanical ventilation requirement, nausea, vomiting, apnea, urinary retention, need for readmission, and length of hospital stay.

## Results

The study patients were divided into two groups based on anesthesia type. The first group included pediatric hernia repair cases performed under general anesthesia (GA group), while the second group included cases of pediatric hernia repair done under combined regional and general anesthesia (GA+RA). The means and proportions of the study participants were calculated to characterize them overall and in groups. The two groups (GA and GA+RA) were compared using the Chi-square or Fisher exact test for categorical factors and the t-test or Kruskal-Wallis test for continuous variables as appropriate. The group comparisons were done in terms of demographic data, peri-operative variables, and outcome variables. The level of significance was declared at α = 0.05. Statistical analysis was conducted using SAS 9.4 (SAS Institute Inc., Cary, NC, USA).

During the study period, 347 children who underwent inguinal hernia repair were screened. Out of the 347 children, 212 fulfilled the study criteria, with 57 patients in the GA group and 155 in the GA+RA group. Their demographic data were summarized in Table 1. Demographic data were comparable between both groups except for age, which was 60.3±49.4 months in the GA group versus 26.7±33.1 months in the GA+RA group (p<.0001). In the group who had general anesthesia alone (N=57), patient and physician preferenece, technique of surgery, and patient requirement were the main factors for selecting the type of anesthesia as summarized in Table 2. Outcome variables demonstrated significant differences by univariate analysis with less postoperative pain occurrence, shorter hospital stay, less bradycardia, and less mechanical ventilation requirement in the GA+RA group in comparison to the GA group with P values of 0.031, 0.02, 0.005, 0.02, respectively (Table 3).

**Table 1:** Comparison of demographic data between general anesthesia group versus general plus regional anesthesia group

Demographic Data	General anesthesia (GA group) N=57	General and regional anesthesia (GA+RA) group N=155	P Value
Gender: Female	16	35	0.51
Mean Age ± SD (months)	60.33±49.4	26.2±32.9	0.0001

**Table 2:** Reason for choosing general anesthesia for pediatric hernia repair.

Reason for general anesthesia technique	General anesthesia (GA group) N=57
Patient and doctor preference	33
Laparoscopic surgery	12
Patient requirement (preterm, vertebral deformity, back anomaly)	12

**Table 3:** Comparison of outcome variables between general anesthesia group versus general plus regional anesthesia group. PACU: post anesthesia care unit.

Outcome Variables	General anesthesia (GA group) N=57	General and regional anesthesia (GA+RA) group N=155	P Value
Post operative Pain	11/57 (19%)	12/155 (8%)	0.031
Post operative analgesic requirement	15/57 (18%)	29/155 (19%)	0.3
Readmission	8/57 (12%)	9/155 (6%)	0.09
Urinary retention	0/57 (0%)	2/155 (1%)	0.95
Incidence of apnea	3/57 (5%)	1/155 (1%)	0.1
Vomiting	4/57 (7%)	9/155 (6%)	0.99
Nausea	0/57 (0%)	4/155(3%)	0.51
Scrotal edema	3/57 (5%)	2/155 (1%)	0.23
Bradycardia	4/57 (7%)	0/155 (0%)	0.005
Tachycardia	3/57 (5%)	4/155 (3%)	0.27
Desaturation and mechanical ventilation requirement	7/57 (12%)	5/155 (3%)	0.02
Hypotension	2/57 (4%)	0/155 (0%)	0.12
Behavioral changes	3/57 (5%)	1/155 (1%)	0.1
Length of stay in PACU (hours)	2.5±9.1	1.2±0.85	0.09
Length of stay in hospital (hours)	70.6±295.3	16.3±47.3	0.02

## Discussion

Our study explored the postoperative benefits of adding regional anesthesia to general anesthesia in pediatric patients undergoing inguinal hernia repair. The option of choosing general anesthesia versus regional plus general anesthesia may result in a better clinical outcome or financial benefits for the patients. In this study, we found advantages in adding regional anesthesia as compared to general anesthesia alone in terms of postoperative pain, bradycardia, desaturation incidence post-operatively, and length of hospital stay.

In our study, we observed that the mean age of patients who had general anesthesia (60.3±49.4 months) was older than that of those who received the combination approach (26.7±33.1 months). This variation was attributed to three primary factors: the patients' choice for general anesthesia alone due to the misconception of the parents regarding spinal injections (33/57), laparoscopic surgery (12/57), and patient requirement, such as prematurity, vertebral deformities, and spinal abnormalities (12/57). On the other hand, individuals with GA and RA combined tended to be younger and comorbidity-free.

We noted in our study that patients who had general and regional anesthesia had a considerably reduced risk of postoperative bradycardia (0%) compared to the general anesthesia group (7%) (P=0.005). A recent meta-analysis of newborns following inguinal hernia surgery found that bradycardia was more common when general anesthesia was utilized (6%) than when combined general and regional anesthesia (3%) [13]. Lower rates of bradycardia and desaturation may be related to reduced systemic analgesic and narcotic needs with the combination of general and regional anesthetic [14]. Additionally, the combination of general and regional anesthesia may lead to an overall decrease in the intake of drugs required to correct bradycardia or any other complications associated with systemic anesthesia.

In terms of hospital stay, a study conducted by Rafiq et al. in 2016 reported that patients with the combined GA+RA technique had a maximum hospital length of stay of two days in comparison to three to four days with general anesthesia alone [15]. In our research, the hospital stay for patients undergoing inguinal hernia repair decreased from 70.6±295.3 hours in the GA group to 16.3±47.3 hours in GA+RA. This 77% reduction in hospital stay suggests that a combined technique may have a better advantage in terms of shorter hospitalizations with less bed occupancy. On the other hand, while financial benefits were not measured directly in this study, a shorter length of stay in the hospital potentially reduces healthcare costs.

It has been reported that 25% of patients undergoing hernia repair suffer from postoperative pain with greater frequency than previously thought [16]. Although the objective assessment of pain in children is difficult, its management following pediatric inguinal hernia repair may include adequate intraoperative analgesia, intraoperative regional anesthesia, and postoperative pain control using opioids, non-steroidal anti-inflammatory medications, and acetaminophen. In a recent meta-analysis on neonates after inguinal hernia repair, postoperative pain was significantly lower in patients who had central and regional anesthesia (odds ratio [OR] 0.44 [95% CI 0.31 to 0.63], I2 = 0%, p<0.001) [17]. In another study, the authors compared three types of analgesia techniques, including general, regional, and local anesthesia, for postoperative pain management. They found that 36%, 0%, and 6% of patients required extra analgesic administration, respectively [18]. Similarly, our study demonstrated a lower prevalence of postoperative pain (8%) in the combined technique in comparison to 19% in the GA group. The combined regional and general anesthesia cost benefit over other types of anesthesia justifies its use in hernia surgery. 

There are published reports that document favorable outcomes with combined regional and general anesthesia techniques for postoperative hernia repair. These include postoperative analgesia needs, apnea, vomiting, nausea, hypotension, and behavioral changes [5,19,20]. In a published meta-analysis, the authors reported a significant difference in postoperative nausea (P=0.045) and vomiting (P=0.034) when comparing regional to general anesthesia for pediatric hernia repair [5]. In addition, a recent study conducted on 394 children who had hernia repair found that among 168 patients exposed to general anesthesia, 44 children (26.2%) developed behavioral abnormalities compared to 12 out of 226 patients (5.3%) who were exposed to regional anesthesia (P< 0.0001). The authors concluded that exposure to anesthesia before the age of two years increases the risk of developing behavioral disorders when surgery is accompanied by general anesthesia [19]. In our study, we noted a trend in behavior changes with general anesthesia cases that did not reach statistical significance, possibly due to the small sample size. Additionally, a multi-center trial on children undergoing inguinal hernia repair published in 2017 found that hypotension occurred more in the general anesthesia group compared with the regional anesthesia group (relative risk, 2.8, 95% CI, 1.7-4.4 by ITT) [18]. Furthermore, a recent meta-analysis published in 2017 comparing episodes of apnea between general anesthesia and regional plus general anesthesia showed that in the regional group, 9% (95% CI: 0.05 to 0.16) of the patients suffered from apnea, whereas 20% (95% CI: 0.15 to 0.27) of the patients in the general group experienced apnea (49 of 241) [21].

## Conclusions

Our study demonstrated that using regional and general anesthesia techniques rather than general anesthesia alone has favorable postoperative outcomes and improved postoperative care in terms of a decrease in postoperative pain, length of hospital stay, bradycardia, and mechanical ventilation requirement. However, further studies are needed to confirm our results.
